# Characterization of Infant Feeding Practices and Related-Family Characteristics in the French Nationwide ELFE Birth Cohort

**DOI:** 10.3390/nu13010033

**Published:** 2020-12-24

**Authors:** Aurore Camier, Claire Chabanet, Camille Davisse-Paturet, Elea Ksiazek, Sandrine Lioret, Marie-Aline Charles, Sophie Nicklaus, Blandine de Lauzon-Guillain

**Affiliations:** 1 Université de Paris, CRESS, Inserm, INRAE, F-75004 Paris, France; camille.davisse-paturet@inserm.fr (C.D.-P.); sandrine.lioret@inserm.fr (S.L.); marie-aline.charles@inserm.fr (M.-A.C.); blandine.delauzon@inserm.fr (B.d.L.-G.); 2 Centre des Sciences du Goût et de l’Alimentation, AgroSup Dijon, CNRS, INRAE, Université Bourgogne Franche-Comté, F-21000 Dijon, France; claire.chabanet@inrae.fr (C.C.); elea.ksiazek@inserm.fr (E.K.); sophie.nicklaus@inra.fr (S.N.); 3 Unité mixte Inserm-Ined-EFS ELFE, Ined, F-75020 Paris, France

**Keywords:** infant feeding practices, family characteristics, cohort, infant feeding patterns

## Abstract

Family characteristics such as education level or income are related to infant feeding practices. This study aimed to characterize infant feeding practices and investigate their associations with family characteristics. Analyses were performed with data from a French nationwide cohort, Etude Longitudinale Française depuis l’Enfance (ELFE). Feeding practices were characterized by two methods, a principal component analysis and a hierarchical ascendant classification (*n* = 8922). This characterization was conducted in three steps: considering firstly only introduction of main food groups, then also food pieces and finally adding the type of complementary food. The associations between family characteristics and the infant feeding patterns or clusters were tested by linear or multinomial regressions (*n* = 7556). Besides breastfeeding duration and age of first introduction of complementary foods, it appeared also important to consider specific food groups such as sweetened beverages and cow’s milk, and the introduction of food pieces, to describe feeding practices. Recommended feeding practices (longer breastfeeding, complementary food in the right period) were related to higher maternal age and education level, so was migration status, the presence of older children, low income or the mothers’ attendance to pre-birth preparation classes. The interrelations between feeding practices and family characteristics must be considered when examining the influence of feeding practices on child’s health.

## 1. Introduction

The first months of life are a critical and sensitive period for later health status; during this time window, several exposures (including diet) represent opportunities to foster a healthy growth and development for children [1]. For example, the benefits of breastfeeding on infection and infant mortality prevention, and neurodevelopment, are well established [2]. However, the diet of non-breastfed infants is not often accounted for, limiting the interpretation of findings for some outcomes [2]. Therefore, a more comprehensive knowledge of early feeding practices is of great importance to identify predictors of later health status in the population.

Infant feeding is characterized by a first period of exclusive milk feeding, with exclusive breastfeeding recommended for the first 6 months or at least 4 months of life [3,4]. Then follows a complementary feeding (CF) period, during which other food groups are gradually introduced, complementing the continued provision of milk. Current recommendations state that the introduction of CF should neither start before 4 months of age nor after 6 months of age [5]. Because of the temporal superimposition of milk and CF, some studies have found strong associations between breast feeding (BF) duration and age at CF introduction [6,7,8]. However, CF practices are defined by the timing and the content but also by the order of introduction of the different food groups, the introduction of food pieces, that have been related to food diversity later in life [9], and the type of food (organic or conventional, home-made or commercial). The associations between all aspects of feeding during this period have yet to be analyzed in detail. In fact, only few studies have considered the full diet before age 1 year (and none before age 6 months) [10].

Family characteristics are known to be associated with infant feeding practices. For example, in high-income countries, BF is associated with high socioeconomic position [11,12,13]: women with high education levels are more likely to initiate BF and breastfeed for longer than others [2,14,15]. CF practices also vary according to demographic factors and migration status: for instance, as compared with older non-immigrant parents, young mothers or migrant parents are more likely to initiate CF before the recommended age of 4 months [8]. Socioeconomic position refers to numerous characteristics and may be linked to a wide range of exposures and susceptibilities [16] which support the need to consider several variables. For example, for young adults, education level captures the long-term effects of both early life factors (resources of the family during childhood) and adult resources (via employment status or health literacy, which allows for being more receptive to health education messages) on health [16]. However, during the life course, household income captures access to health services and better-quality material resources such as food or housing.

In this context, this work aimed first to characterize infant feeding practices during the first year in a high-income country (France) by using two complementary methods (i.e., principal component analysis and hierarchical ascendant classification) and examining the complexity of diet in three steps: considering firstly only the introduction of the main food groups, then also the introduction of food pieces and finally the type of food (organic, or ready-prepared baby food). This work also aimed to investigate the associations between family characteristics and the identified infant feeding practices patterns or clusters.

## 2. Methods

### 2.1. Study Design and Setting

The ELFE study (Etude Longitudinale Française depuis l’Enfance) is a multidisciplinary nationwide birth cohort including 18,329 children born in 2011 in a random sample of 349 maternity wards from mainland France [17]. The cohort aims at studying the determinants of child development, health and socialization from birth to adulthood. Inclusion began in April 2011 and took place during 25 selected recruitment days over four waves of four to eight days each that covered all four seasons. Inclusion criteria were as follows: singleton or twins born after 33 weeks of gestation, to mothers aged 18 years or older and not planning to move outside of metropolitan France in the next 3 years.

Participating mothers had to provide written consent for their own and their child’s participation. When present at inclusion, fathers signed the consent form for the child’s participation or were informed about their rights to oppose it. The ELFE study was approved by the Advisory Committee for Treatment of Health Research Information (Comité Consultatif sur le Traitement des Informations pour la Recherche en Santé), the National Data Protection Authority (Commission Nationale Informatique et Libertés), and the National Statistics Council.

### 2.2. Variables

#### 2.2.1. Infant Feeding Practices

Milk feeding practices were collected at 2, 12 and 24 months by phone interview and monthly from 3 to 10 months by an Internet-based or paper questionnaire. During each follow-up step, current feeding mode was recorded: human milk only, formula milk only, both human and formula milk, cow’s milk or plant-based beverages (almond-based beverage, rice-based beverage, soya-based beverage, excluding infant formulas based on rice or soya). At each phone interview, whenever the mother had stopped breastfeeding, the exact age of the child when breastfeeding had ended was asked along with the age of introduction of formula milk. From these data, any BF duration and age at infant formula introduction were calculated. The exact age when the mother stopped breastfeeding was used to calculate the duration of any breastfeeding; if it was missing, the age at the completion of the last questionnaire indicating current breastfeeding was considered. The detailed method used was previously described [18]. Any BF duration and age at infant formula introduction were considered as continuous variables. Details on milk feeding practices were collected monthly from 3 to 10 months post-partum, using an online or paper self-reported questionnaire. For the 3- to 10-month questionnaire, parents reported the usual number of bottle- and breastfeeds, as well as the usual average quantity of a bottle, when relevant, consumed during a typical day of the current month.

Infants’ CF consumption was also collected with the 3- to 10-months questionnaire. This questionnaire consisted of 26 food items (fruit juices, other sweetened beverages, regular cow’s milk, semi-skimmed or skimmed cow milk, infant cereals, potatoes, green beans, carrots, peas, artichokes, other vegetables, pasta/rice, bread, meat, fish, egg yolks, egg whites, cheese, yogurt, apples, bananas, strawberries, peaches, other fruits, biscuits, and other desserts). The frequency of their consumption during the last month was recorded on a 5-point scale: not yet introduced (0), introduced once (1), several times (2), often (3), and every day or almost every day (4). From these data, the age at introduction of 8 food groups (fruits and vegetables, potatoes, sweetened beverages and fruit juices, baby cereals, meat/fish/eggs, bread/pasta/rice, dairy products, cow’s milk) was calculated, as previously described [8]. A food group was considered introduced if it was eaten at least twice by the infant. The age at introduction of food pieces (e.g., foods crushed by a fork or in pieces) was also calculated from the consumption of crushed vegetables or fruits (collected from the third month on) and pieces of meat (collected from the sixth month on).

For each food group and for food pieces, an indicator variable for the frequency of consumption over the first year of life was calculated using the median of frequencies declared each month (1 = introduced only once, 2 = several times, 3 = often, 4 = every day or almost every day, 5 = several times per day), starting from the month of introduction of the food group.

Each month, the parents reported the type of foods used during CF—organic foods and ready-prepared baby foods—by using a 4-point Likert scale (0 = never, 1 = sometimes, 2 = often, 3 = almost always). The choice of organic foods or commercial complementary foods for CF were summarized as the modal-reported frequency of their use between the age of introduction to complementary food and age 10 months.

#### 2.2.2. Family Characteristics

Maternal and household data were collected by face-to-face interviews at the maternity ward and then by phone interview at the 2-month follow-up. Family characteristics collected during the maternity stay were used only when the 2-month values were missing.

Family characteristics included in the analyses were maternal age (<25, 25–29, 30–34, ≥35 years), maternal education level (up to lower secondary, upper secondary, intermediate, 3-year university degree, at least 5-year university degree), family income by monthly consumption unit (up to €750, €751–1111, €1112–1500, €1501–1944, €1945–2500, >€2500), maternal migration status (migrant, descendant of migrant, majority of population), single motherhood (yes/no), older children in the household (no older child, one older child, at least two older children), child’s sex (female/male), and projected maternal return to work (not employed during pregnancy, <10 weeks, 10–13 weeks, 3–6 months, after 6 months). A second group of family characteristics considered as health-related variables were maternal smoking status during pregnancy (never smoker, smoker only before pregnancy, smoker only in early pregnancy, smoker throughout pregnancy), maternal pre-pregnancy body mass index (BMI) (<18.5, 18.5–24.9, 25–29.9, ≥30 kg/m^2^), first physician consulted for the child after hospital discharge (pediatrician, other child doctor, general practitioner, none/other), attendance at pre-birth preparation classes (none, <7, ≥7).

Infant characteristics used only for population selection were gestational age at birth (<37 or ≥37 weeks’ gestation) and twin pregnancy (yes, no).

### 2.3. Sample Selection

From the 18,329 infants included in the ELFE study, children for whom parents withdrew consent were excluded from the study, as were preterm (<37 weeks’ gestation) or twin babies because of specific dietary issues (*n* = 1317, including 57 consent withdrawal), which resulted in 17,012 eligible infants. We excluded infants with missing data on infant feeding practices (*n* = 8090), which resulted in 8922 infants for characterization of infant feeding practices. We excluded infants with a least one missing data on family characteristics (*n* = 1366), which resulted in 7556 infants for complete-case analysis of the association between family characteristics and infant feeding practices.

### 2.4. Statistical Methods

Comparisons between excluded and included subjects involved chi-square tests for categorical variables and Student *t* test for continuous variables. Analyses were conducted by using SAS v9.4 [19].

#### 2.4.1. Infant Feeding Practices Typologies

Infant feeding practices were summarized with two complementary approaches: principal component analysis (PCA) and hierarchical ascendant classification (HAC). They represent the two main approaches used in the literature in this topic [6,10,20,21]. The identification of infant feeding patterns by PCA describes correlations between feeding practices. The PCA method facilitates a more balanced individual classification because each subject has a score on each pattern. By construction, the different patterns are independent of each other, which facilitates the identification of a pattern-specific effect. In contrast, HAC classifies infants in clusters who received similar feeding practices. The HAC method allows for identifying mutually exclusive sub-groups and then an easier description of those sub-groups. Indeed, each cluster of infants may be described as any classical population description.

The number of dimensions identified from the standardized PCA was determined by using the distribution of eigen values and the interpretation of patterns. Variables with factor loading >|0.4| in absolute value were considered to contribute significantly to the pattern and were used to interpret the pattern. All subjects have a score on each dimension retained. This score can be interpreted as a subject’s compliance with the pattern, with a high score indicating high adherence.

The HAC was conducted with the Ward’s clustering method on Euclidean distances after standardization of the same sets of variables as those used for PCA analyses, and up to 10 clusters were considered. The number of clusters was guided by three criteria: cubic clustering criterion, pseudo F, and pseudo T^2^. Clusters were named according to the distribution of the feeding practices most characteristic of each cluster. One single variable with as many classes as identified clusters was constructed indicating the cluster membership.

For each approach, three different sets of variables were used. First, the analysis focused on 20 variables usually collected in studies on infant diet (Set A variables): any BF duration, daily number of breastfeeds at the age of CF initiation, age at introduction of infant formula, daily number of bottles at the age of complementary food initiation, age at introduction and consumption frequency indicator after introduction for each of the eight food groups previously presented (fruits and vegetables, potatoes, sweetened beverages and fruit juices, baby cereals, meat/fish/eggs, bread and pasta, dairy products, cow’s milk). In a second step, the two variables related to food pieces (age at introduction and consumption frequency indicator after introduction, relative to fruits and vegetables, and meat) were added (Set B variables). In a third set, characteristics related to the type of foods used during CF (organic foods and commercial complementary foods) were considered (Set C variables), as in previous studies [6,22].

#### 2.4.2. Relations between Family Characteristics and Feeding Practices

Associations between family factors and infant feeding practices were tested by simultaneously considering family characteristics (maternal age, educational level, monthly family income, migration status, single motherhood, older children in the household, child’s age at maternal return to work, child’s sex) and variables related to study design (maternity size, wave of recruitment and region of residence). The reference category was the one with the largest number. In a second model, we also considered health-related variables (smoking status during pregnancy, pre-pregnancy BMI, attendance at pre-birth preparation classes, first physician consulted). For infant feeding patterns identified by PCA, we used separated adjusted linear regressions for each pattern. For clusters identified by HAC, we used adjusted multinomial regression.

Two sensitivity analyses were conducted. The first consisted of multiple imputation on family characteristics. The second used weighted data to correct for bias related to inclusion procedure, non-consent and attrition. Weighting also included calibration on margins from the state register’s statistical data and the 2010 French National Perinatal study [23] for the variables age, region, marital status, migration status, level of education, and primiparity. This weighting was calculated for the subsample that completed the questionnaire on infant diet, at least once from 3 to 10 months [24].

## 3. Results

### 3.1. Selected Population

Compared with non-included mothers (*n* = 8849), those included (*n* = 8922) were significantly older (mean: 31 vs. 30 years, *p* < 0.001), less often smoked during pregnancy (12% vs. 21%, *p* < 0.001), had higher education level (25% vs. 14% with at least a 5-year university degree, *p* < 0.001), had lower pre-pregnancy BMI (23.3 vs. 23.7 kg/m^2^, *p* < 0.001), had higher monthly family income (mean €1784 vs. €1436, *p* < 0.001), and had lower number of older children in the household (16% vs. 22% with at least 2 older children, *p* < 0.001) (data not shown).

For the associations with family characteristics, 1366 additional mothers were excluded because of missing data on studied characteristics. As compared with these excluded mothers, included mothers (*n* = 7556) were significantly younger (mean 31 vs. 32 years, *p* = 0.02), had higher education level (26% vs. 31% with upper secondary level, *p* < 0.001), had higher family income (mean €1799 vs. €1675, *p* < 0.001) and had lower number of older children in the household (47% vs. 37% with no older child in the household, *p* < 0.001) (data not shown). The groups did not differ in pre-pregnancy BMI (*p* = 0.26) or proportion of smokers during pregnancy (11.5% vs. 13.9%, *p* = 0.07).

### 3.2. Identification of Infant Feeding Practice Patterns by PCA

When only core variables (BF and age of introduction and frequency of consumption of main food groups; i.e., Set A variables) were considered, we identified six feeding patterns (Table 1), explaining 67% of the total variance. The first pattern was labeled “A1—later CF initiation and longer BF duration”, the second “A2—longer BF and higher BF rate”, the third “A3—frequent intake of main food groups” (fruits/vegetables, potatoes, meat/fish/eggs, dairy products), the fourth “A4—earlier cow’s milk”, the fifth “A5—earlier baby cereals” and the sixth “A6—earlier sweetened beverages and fruit juices”.

When food pieces (Set B variables) were also considered, we identified five feeding patterns (Table 2), explaining 57% of the total variance. The patterns were similar to previous analyses except that the pattern characterized by earlier introduction of sweetened beverages and fruit juices was no longer observed. Indeed, the variables contributing to this sixth pattern were, in this analysis, related to baby cereals consumption. Moreover, we found a modification of the contribution of BF to Patterns 1 and 2, with a lower contribution of BF to Pattern 1 and a higher contribution to Pattern 2, and an opposite contribution of food pieces (positively to Pattern 1 and negatively to Pattern 2). The three subsequent patterns were only marginally modified. The consumption of food pieces was positively related to Pattern 2 and negatively to Pattern 5. These patterns were then relabeled “B2—very long BF, higher breastfeed rate, earlier food pieces” for Pattern 2 and “B5—earlier baby cereals and later food pieces” for Pattern 5.

Finally, when organic and commercial complementary foods were also considered (Set C variables), we identified five feeding patterns (Table 3), explaining 53% of the total variance. Consumption of organic foods or commercial complementary foods was not related to any pattern. The patterns were only marginally modified as compared to previous analyses, and labels remained almost the same.

### 3.3. Clustering of Infants by Feeding Practices Using HAC

When only core variables were considered (Set A variables), we identified five clusters of infants based on feeding practices (Table 1). Two clusters were characterized by intermediate BF but distinguished by intermediate CF (Cluster A1′, *n* = 1916) or late CF (Cluster A2′, *n* = 2726). Intermediate CF refers to a CF started neither early (before 4 months) nor late (after 6 months) according to the current definitions and applicable guidelines. Two clusters were characterized by short BF and early CF but were distinguished by very early introduction of sweetened beverages and fruit juices (Cluster A3′, *n* = 1587) or cow’s milk (Cluster A5′, *n*= 1063). The last cluster was characterized by long BF and intermediate CF (Cluster A4′, *n* = 1630).

When food pieces were also considered (Set B of variables), five clusters of infants were identified (Table 2). As previously described, two clusters were characterized by intermediate BF but distinguished by intermediate CF and no sweetened beverages and fruit juices (Cluster B1′, *n* = 2891) or late CF, late food pieces and infrequent main food groups (Cluster B2′, *n* = 1703). Similarly, two clusters were characterized by short BF and early CF but distinguished by very early introduction of sweetened beverages and fruit juices (Cluster B4′, *n* = 1576) or cow’s milk (Cluster B5′, *n*= 1019). The last cluster was characterized by long BF, intermediate CF and early food pieces (Cluster B3′, *n* = 1733).

Finally, when organic and commercial complementary foods were additionally considered (Set C of variables), five clusters were identified (Table 3). Most clusters remained very similar to the previous clustering. The previous cluster “B4′. Short BF, early CF and very early sweetened beverages and fruit juices” was also related to frequent use of commercial complementary foods in this clustering and relabeled “C4′. Short BF, early CF, very early sweetened beverages and fruit juices, frequent commercial complementary foods”.

To summarize, when food pieces were also considered (Set B variables), 80% of infants were classified in similar clusters as those described for core variables (Set A variables). The main change occurred for the “core variable” cluster characterized by intermediate BF and late CF (Cluster A2′), with 41% reattributed to the Cluster “B1′—intermediate BF and intermediate CF no sweetened beverages and fruit juices” and 55% remaining in the Cluster “B2′—intermediate BF and late CF, late food pieces and infrequent main food groups”. Thus, considering food pieces allowed for better characterization of late CF. When organic and commercial complementary foods were also considered (Set C variables), the clusters only marginally changed, with 88% of infants remaining in similar clusters.

Feeding practice patterns appeared to be precisely characterized when both core variables and food pieces were considered. The Set C variables, with variables related to consumption of organic or commercial complementary foods, seems to contribute weakly to the description. We then considered only the Set B variables patterns or clusters to relate them to family characteristics.

Cluster labels were chosen according to the variable distribution in the cluster relatively to other clusters.

### 3.4. Associations of Feeding Patterns with Familial Characteristics: Multivariable Analysis

Maternal age was positively related to the “B1—later CF and longer BF” pattern, but the association with the “B2—very long BF, higher breastfeed rate and earlier food pieces” and “B3—frequent intake of main food groups” patterns was not monotonous (Table 4). Maternal education level was positively related to the “B1—later CF and longer BF”, “B2—very long BF, higher breastfeed rate and earlier food pieces” and “B3—frequent intake of main food groups” patterns. Family income was negatively related to the “B2—very long BF, higher breastfeed rate and earlier food pieces” pattern but positively to the “B3—frequent intake of main food groups” pattern. Having a migrant mother was positively related to the “B2—very long BF, higher breastfeed rate and earlier food pieces” pattern but negatively to the “B1—later CF and longer BF” and “B3—frequent intake of main food groups” and “B4—earlier cow’s milk” patterns. Single motherhood was negatively related to the “B3—frequent intake of main food groups” and “B4—earlier cow’s milk” patterns. Number of older children was negatively related to the “B3—frequent intake of main food groups” pattern and positively to the “B4—earlier cow’s milk” pattern. As compared with women without older children, those with only one older child were more likely to have high score on the “B1—later CF and longer BF” pattern and low score on the “B5—earlier baby cereals and later food pieces” pattern, whereas those with at least two other children were more likely to have a high score on the “B2—very long BF, higher breastfeed rate and earlier food pieces” pattern. Having a boy was negatively related to the “B1—later CF and longer BF” pattern and positively to the “B5—earlier baby cereals and later food pieces” pattern. Finally, maternal return to work at least 6 months after delivery was positively related to the “B1—later CF and longer BF”, “B2—very long BF, higher breastfeed rate and earlier food pieces” and “B5—earlier baby cereals and later food pieces” patterns.

The previous observations were similar when adding health-related variables in the models (data not shown). Health-related variables were less frequently related to infant feeding patterns than were family characteristics (Table 5). Maternal smoking during pregnancy was negatively related to the “B1—later CF and longer BF” pattern. Maternal overweight (or obesity) before pregnancy was negatively related to the “B1—later CF and longer BF” and “B3—frequent intake of main food groups” patterns. A visit to a pediatrician for the first consultation after hospital discharge was positively related the “B3—frequent intake of main food groups” pattern. Frequent attendance at pre-birth preparation classes was related to an increased score on the “B1—later CF and longer BF”, “B2—very long BF, higher breastfeed rate, earlier food pieces” and “B3—frequent intake of main food groups” patterns.

Values are β [95% confidence interval (CI)] adjusted on familial characteristics (maternal age, maternal education level, family monthly income per consumption unit, migration status, single motherhood, older children in the household, child’s age at maternal return to work, child’s sex) and variables related to study design (region, recruitment wave and maternity size).

BMI, body mass index: regarding the association with clusters, associations between family or health-related variables and the cluster characterized by “B3′—long BF, intermediate CF and early food pieces” as compared with the Cluster “B1′—intermediate BF and intermediate CF” were consistent with those highlighted for Pattern B2 from PCA analysis, characterized by “B2—very long BF, higher breastfeed rate and earlier food pieces”: positive gradient for maternal education level, negative gradient for family income and more likely if the mother is a migrant, with at least 2 older children and a late return to work (or not concerned) (Table 6 and Table 7).

Cluster “B4′-short BF, early CF, very early sweetened beverages and fruit juices” and “B5′—short BF, early CF, very early cow’s milk”, both characterized by short BF and early CF, were in an identical way related to maternal age, maternal education level, pre-pregnancy BMI and child’s sex: related to younger mothers and a male infant, negative gradient for maternal education level, and positive gradient for pre-pregnancy BMI. These associations were in agreement with those highlighted for “B1—later CF and longer BF” but in an opposite way. Clusters B4′ and B5′ were also related to low family income. Migrant or single mother status was related to Cluster B4′ characterized by very early introduction to sweetened beverages and fruit juices. As observed for the “B4—early cow’s milk” PCA pattern, having older children in the household but not single motherhood and maternal migration status was related to belonging to the “B5′—short BF, early CF, very early cow’s milk” cluster.

All associations remained globally similar in the two sensitivity analyses (Supplementary Tables S1–S4) except in the weighted analysis, in which being a single mother was related to belonging to three clusters: “B2′—intermediate BF, late CF, late food pieces, infrequent of main food groups”, “B4′—short BF, early CF, very early sweetened beverages and fruit juices” and “B5′—short BF, early CF, very early cow’s milk“.

## 4. Discussion

To characterize infant feeding practices, our findings highlight that besides BF duration and age of introduction to the main food groups, the introduction of food pieces, sweetened beverages and fruit juices and cow’s milk should be considered. We showed consistencies in feeding practice typologies regardless of the analysis method and variables included. Feeding practices not recommended by current guidelines (early introduction of cow’s milk, sweetened beverages and fruit juices) were less frequent in with older maternal age, higher education level, and later return to work. However, these feeding practices were also less frequent in migrant families, families with older children and low income or when mothers had attended pre-birth preparation classes.

The consistency of results between the two different methods strengthens the validity of our findings. The HAC method allows for characterizing groups of infants with similar feeding practices and providing frequencies in the population of the different infant feeding practices. In addition, the interpretation of its results allows for examining whether non-adherence to the different guidelines are combined: children who are breastfed for a short duration (or not breastfed) appear to also be those with the earliest CF initiation and inappropriate food groups introduction such as early sweetened beverages and fruit juices or cow’s milk. The association between longer BF and later CF initiation is widely described in other studies [8,25,26,27,28]. Hence, this method appears adequate to identify and precisely describe at-risk sub-groups of infants and investigate potential associations with these combinations of practices. However, distinguishing the specific effect of early cow’s milk introduction (or very early sweetened beverage introduction for the other cluster) could be difficult in further association studies (e.g., health implications). The PCA method facilitates more powered analyses because each subject has a score on each pattern. Moreover, each infant has a score on each pattern and the different patterns are independent of each other. This characteristic of the PCA method could be attractive if, for example, the feeding practice characterizations were used as adjustment factors. In the present analyses with the PCA method, BF duration and CF introduction were the main parts of Patterns 1 and 2 but did not contribute to patterns “A4- earlier cow’s milk” or “A6- earlier sweetened beverages and fruit juices”. Consequently, the independent effect of these latter practices can be examined. Thus, the two approaches are complementary, depending on the objective of the analysis.

The Avon Longitudinal Study of Parents and Children (ALSPAC), a birth cohort recruiting pregnant women between 1991 and 1992, identified four dietary patterns at 6 and 15 months [22]. Nevertheless, because of the methodological differences between the studies, we cannot compare these dietary patterns to our feeding practices patterns or clusters. Moreover, at this time, recommendations about complementary feeding were different. The ALSPAC “biscuits, sweets and crisps” patterns at 6 and 15 months were similar to feeding practices identified in the ELFE study, especially those characterized by early sweetened beverages and fruit juices introduction (Pattern A6 and Clusters A3′, B4′, C4′) because their patterns were associated with increased consumption of sugar added to bottles at 6 months and increased consumption of cola and other fizzy drinks at 15 months. Moreover, the ALSPAC “breastfeeding” pattern may be compared to feeding practices characterized by longer BF duration in the present study, despite the absence of information about age at introduction of complementary food in their pattern characterization.

Regarding the Etude des Déterminants du développement et de la santé de l’Enfant (EDEN) mother–child cohort [6], the methods used were closer to those used in this paper. Three infant feeding practice patterns were identified. The one characterized by longer BF, later complementary food initiation and home-made foods was very close and comparable to patterns or clusters characterized by long BF duration and late (or intermediate) complementary food introduction (Patterns A1, B1, C1 and Clusters A4′, B3′, C2′) in our study. Unfortunately, data on food pieces, cow’s milk or sweetened beverages and fruit juices introduction were not available in the EDEN mother–child cohort and those on home-made foods were not available in the ELFE study.

With data from the Infant Feeding Practices Study II (IFPS II) in the United States in 2005 [29], Rose et al. found five infant dietary patterns by using latent class analysis. Two BF and two formula-feeding classes were identified and differed in the variety of solid foods at 9 months (low or high variety fruits and vegetables). Those four classes are not very comparable to our results. It could be explained by different feeding practices at the country level, different methods used, or different periods studied (2005 versus 2011). However, the fifth class (characterized by increased likelihood of feeding with cow’s milk and energy-dense items including sweet drinks, juices, sweet foods, French fries and a combination of both breastmilk and formula milk) shows similarities with some of our clusters such as “B4′—short BF, early CF, very early sweetened beverages and fruit juices” or “B5′—short BF, early CF and very early cow’s milk” (and equivalent clusters in the other analyses). Indeed, the IFPS II fifth class combines unhealthy feeding practices: early consumption of cow’s milk or sweetened drinks and short exclusive BF duration. In the ELFE study, early cow’s milk introduction was combined with a global earlier introduction of complementary food. In line with this finding, in the Longitudinal Study of Child Development in Quebec, Canada [30], 82% of the children who received complementary food before 4 months also received cow’s milk before 9 months.

Overall, the consideration of food pieces allowed for better distinguishing the pattern related to CF and the pattern related to BF. The food pieces introduction seems associated with other feeding practices such as age of introduction of any complementary food or home-made food for example [31]. Indeed, a French study showed strong associations between food texture exposure and other feeding practices such as introduction of complementary food (negative associations), food preparation type (negative associations with ready-prepared baby food) or BF (inconsistent associations) [32]. Moreover, in the ALSPAC study, authors found that a later introduction of lumpy foods (after 9 months) was associated with lower diversity in diet at seven years, along with more feeding problems compared to an earlier introduction of lumpy foods [9]. Considering the timing of food pieces introduction is therefore of great importance to better understand the influence of early feeding practices on later diet. Conversely, the consideration of organic and commercial complementary foods did not substantially change the feeding pattern characterization because of their homogenous distribution into clusters and patterns. To summarize, simultaneously considering BF variables (duration and number of breastfeeds), main food group variables (age of introduction and consumption frequency of fruits and vegetables, potatoes, sweetened beverages and fruit juices, baby cereals, meat/fish/eggs, bread and pasta, dairy products, cow’s milk) and food pieces variables appears to represent the best compromise to characterize infant feeding practices in this study (the French context).

Studies in high-income countries reported that both early complementary food introduction (before age 4 months) and short BF duration were more frequent in socioeconomic less-advantaged families often characterized by low maternal age (<30 years old), low education level, and smoking and unemployment status [7,8,14,30,33,34,35]. In the present study, regardless of the method used to characterize feeding practices (PCA or HAC), the combination of short BF and early CF introduction was more frequent in families with low socioeconomic status: low maternal education level, young maternal age, and smoking during pregnancy. More widely, a systematic review highlighted positive associations between older maternal age or high maternal education level and “healthy” dietary patterns up to 2 years [10]. In this review, “healthy” patterns were defined in accordance with the World Health Organization international dietary recommendations for children’s age and designated as “conscious health pattern or Mediterranean diet” or “breastfeeding or milk formula with fruits and vegetables” for example. In contrast, “unhealthy” referred to “Western-like pattern” or “high sugar, fat, and protein food pattern” for example. “Unhealthy” feeding practices were associated with low maternal age and low education level in several birth cohorts: in France (EDEN), with early introduction of fruit juices associated with lower maternal age [6]; in England (ALSPAC), with consumption of biscuits or sweetened beverages related to low maternal age and education level [22]; and in the United States (IFPS II), with early consumption of cow’s milk, sweet foods and drinks associated with low education level [29]. This observation is consistent with the present findings of early introduction of sweetened beverages and fruit juices or cow’s milk.

For family income, findings are less consistent across the literature. Some studies did not highlight an association between early feeding practices and family income [6,29], but a recent systematic review reported a positive association between BF maintenance after 12 months and low family income [35]. In accordance with this review, long BF duration was related to low family income in the ELFE study. However, early introduction of sweetened beverages was more frequent in less advantaged households [10], which could probably explain the association we found between low family income and belonging to clusters characterized by short BF duration, early CF and very early introduction of sweetened beverages (Clusters A3′, B4′, C4′).

Some family characteristics were less often studied in previous reports. A systematic review reported a positive association between being a migrant or foreigner and BF maintenance for 12 months or more [35], which is consistent with our results. In the ELFE study, migrant mothers frequently introduced earlier complementary food while maintaining long BF [8]. Moreover, multiparous women frequently initiated and maintained BF for a longer time [35] but also adopted some feeding practices not recommended by current guidelines [6,10,22], as we found for early cow’s milk introduction. Maternal health-related characteristics were weakly associated with feeding practices, but the attendance at pre-birth preparation classes and consulting a pediatrician after hospital discharge were related to healthy feeding practices. These latter associations might be explained by advices given by health care providers but also might reflect a greater health consciousness of women who consulted these health care providers. The World Health Organization, through the Baby Friendly Hospital Initiative, recommends antenatal information [36] as one of the 10 steps to increase initiation and continuation of BF. Our study suggests that it could also help encourage healthy CF practices. Moreover, a recent meta-analysis identified “breastfeeding education” as one of the six factors (including mode of delivery, education level or infant–mother separation) positively associated with initiation and continuation of BF [15]: antenatal classes were related to increased rates of BF. Moreover, as in the ELFE study, women who did not attend pre-birth preparation classes were at increased risk of less optimal compliance with recommendations about the introduction of CF [33].

The ELFE cohort is a nationwide birth cohort (excluding very premature babies) established in 2011 in metropolitan France. The main strengths of the ELFE study include the large sample and the wide range of socio-demographic variables and profiles. Moreover, data collection from 2 to 10 months was prospective, which allowed for limiting the memory bias regarding infant feeding. Unfortunately, the prospective nature leads to difficulties comparing our results to other settings. As the use of water, water-based drinks and fruit juice was not collected before the 3-to-10-month questionnaire, we were not able to examine exclusive breastfeeding according to the WHO definition. Therefore, we used any breastfeeding duration and age at infant formula introduction to characterize milk feeding during infancy. In addition, the 3-to-10-month questionnaire was not validated, which may reduce the accuracy of the data collected. The patterns and clusters identified to characterize infant feeding practices were labelled to summarize information. Even if, efforts were made to base these labels on variables distribution or leading factors, we cannot exclude a part of subjectivity in labeling. Nevertheless, those two methods are widely used in nutritional epidemiology. The main analyses were conducted on the complete-case sample, but when missing data on family characteristics were addressed by a multiple imputation method, results remained consistent. Moreover, the sample considered for the present analysis was based on more socio-economically advantaged families than the initial ELFE sample, which limits the generalizability of our results. However, to overcome this issue, sensitivity analysis based on weighted data helped to deal with selection and attrition bias and produced similar findings, which suggests that these biases had limited impact on our results.

In this French nationwide birth cohort, the present study highlighted that besides breastfeeding duration and complementary food introduction, some other aspects of complementary feeding need to be consider to have a more comprehensive view of infant feeding practices: the introduction of specific food groups such as cow’s milk and sweetened beverages, but also the introduction of food pieces which represents an important step of complementary feeding. PCA and HAC produced consistent findings and the choice of the method has to be driven by the need to distinguishing groups of children with homogeneous practices (by HAC) or to examine the independent effect of some practices (by PCA). Our finding also confirmed that higher income and education level were associated with healthier feeding practices, but also highlighted the potential role of some factors in establishing healthy feeding practices such as the attendance at pre-birth preparation classes or the presence of older children in the household. These characteristics could be considered to develop intervention aimed at promoting healthy infant feeding practices. Finally, both the interrelations between the feeding practices and their strong associations with family characteristics must be considered when examining the effect of early feeding practices on a child’s health and development.

## Figures and Tables

**Table 1:** Analysis of core variables (breast feeding (BF) and main food groups), Set A of variables (*n* = 8.922).

	Principal Component Analysis (PCA)						Hierarchical Ascendant Classification (HAC)				
	Pattern 1	Pattern 2	Pattern 3	Pattern 4	Pattern 5	Pattern 6	Cluster 1	Cluster 2	Cluster 3	Cluster 4	Cluster 5
% variance explained (PCA) or % (*n*) (HAC)	21%	15%	10%	8%	7%	6%	21% (1916)	31% (2726)	18% (1587)	18% (1630)	12% (1063)
Any BF duration (mo)	0.62	0.66	−0.06	−0.06	0.11	−0.03	1.4 [0;3.8]	1.9 [0;4]	0.7 [0;2.7]	10 [7;14]	0.9 [0;3]
Introduction * (mo)—Infant formula	0.62	0.62	−0.01	−0.07	0.11	−0.04	0.7 [0;2]	0.9 [0;2]	0.1 [0;1.1]	6 [4;9]	0.2 [0;1.5]
Introduction (mo)—Baby cereals	0.59	0.04	0.27	0.19	−0.63	0.08	6 [5;8]	12 [7;12]	6 [5;7]	9 [7;12]	6 [5;8]
Introduction (mo)—Fruits/vegetables	0.58	−0.23	0.10	0.07	0.22	0.32	6 [5;6]	6 [5;6]	5 [5;6]	6 [5;6]	5 [5;6]
Introduction (mo)—Potatoes	0.53	−0.30	−0.14	0.04	0.18	0.29	6 [6;7]	7 [6;7]	6 [5;7]	6 [6;7]	6 [5;7]
Introduction (mo)—Meat/fish/eggs	0.58	−0.27	−0.15	0.09	0.20	0.24	7 [7;8]	8 [7;8]	7 [6;7]	7 [7;8]	7 [6;8]
Introduction (mo)—Dairy products	0.58	−0.26	0.03	0.00	0.14	0.17	7 [6;8]	7 [7;9]	7 [6;7]	7 [6;8]	6 [6;7]
Introduction (mo)—Bread/pasta/rice	0.43	−0.43	0.10	0.15	0.06	0.35	8 [7;9]	9 [8;12]	8 [7;9]	8 [7;9]	8 [7;9]
Introduction (mo)—Sweetened beverages & fruit juices	0.44	−0.32	0.39	0.35	0.21	−0.52	12 [12;12]	12 [12;12]	7 [5;9]	12 [7;12]	10 [7;12]
Introduction (mo)—Cow’s milk	0.31	−0.31	0.34	−0.76	−0.04	−0.10	12 [12;12]	12 [12;12]	12 [12;12]	12 [12;12]	9 [7;10]
Number of breast feeds at CFI	0.50	0.74	−0.11	−0.09	0.11	−0.06	0 [0;0]	0 [0;0]	0 [0;0]	5 [4;7]	0 [0;0]
Number of bottle feeds at CFI	−0.54	−0.66	−0.01	0.02	−0.11	0.06	4 [4;4]	4 [4;4]	4 [4;5]	0 [0;1]	4 [4;4]
Frequency * (0–4)—Baby cereals	−0.47	−0.05	−0.14	−0.17	0.79	−0.02	4 [2;4]	0 [0;2]	4 [2;4]	2 [0;3]	3 [2;4]
Frequency (0–5)—Fruit/vegetables	−0.24	0.21	0.63	0.06	0.12	0.27	5 [5;5]	5 [4;5]	5 [4;5]	5 [4;5]	5 [4;5]
Frequency (0–4)—Potatoes	−0.18	0.25	0.59	0.10	0.11	0.21	3 [3;3]	3 [2;3]	3 [2;3]	3 [2;4]	3 [2;3]
Frequency (0–4)—Meat/fish/eggs	−0.28	0.21	0.63	0.06	0.06	0.22	3 [3;4]	3 [2;4]	3 [3;4]	3 [3;4]	3 [3;4]
Frequency (0–4)—Dairy products	−0.35	0.18	0.38	0.09	0.03	0.16	4 [3;4]	3 [2;4]	4 [3;4]	3 [2;4]	4 [3;4]
Frequency (0–4)—Bread/pasta/rice	−0.29	0.39	0.16	−0.09	0.04	−0.15	2 [2;3]	2 [0;3]	2 [2;3]	2 [2;3]	2 [2;3]
Frequency (0–4)—Sweetened beverages & fruit juices	−0.39	0.32	−0.38	−0.35	−0.18	0.58	0 [0;0]	0 [0;0]	2 [2;2]	0 [0;2]	1 [0;2]
Frequency (0–4)—Cow’s milk	−0.29	0.29	−0.33	0.78	0.06	0.12	0 [0;0]	0 [0;0]	0 [0;0]	0 [0;0]	2 [2;3]

Data are factor loading for the PCA and median [Q1–Q3] for the HAC. * Introduction: introduced more than once. Frequency: median frequency after introduction. Pattern 1 was labelled “A1—later CF, long BF”; Pattern 2 “A2—longer BF and higher BF rate”; Pattern 3 “A3—frequent intake of main food groups”; Pattern 4 “A4—earlier cow’s milk”; Pattern 5 “A5—earlier baby cereals”; Pattern 6 “A6—earlier sweetened beverages and fruit juices”. Cluster 1 was labelled “A1′—intermediate BF, intermediate CF”; Cluster 2 “A2′—intermediate BF, late CF”, Cluster 3 “A3′—short BF, early CF, very early sweetened beverages”, Cluster 4 “A4′—long BF, intermediate CF” and Cluster 5 “A5′—short BF, early CF and very early cow’s milk”. BF, breastfeeding; CF, complementary feeding. CFI, complementary food introduction.

**Table 2:** Analysis of core variables (BF and main food groups) and food pieces, Set B of variables (*n* = 8922).

	Principal Component Analysis (PCA)					Hierarchical Ascendant Classification (HAC)				
	Pattern 1	Pattern 2	Pattern 3	Pattern 4	Pattern 5	Cluster 1	Cluster 2	Cluster 3	Cluster 4	Cluster 5
% variance explained (PCA) or % (*n*) (HAC)	20%	15%	9%	7%	6%	32% (2891)	19% (1703)	19% (1733)	18% (1576)	11% (1019)
Any BF duration (mo)	0.52	0.74	−0.01	−0.03	0.16	1.4 [0;3.8]	1.9 [0;4.8]	9.8 [7;14]	0.7 [0;2.7]	0.7 [0;2.8]
Introduction * (mo)—Infant formula	0.53	0.69	0.03	−0.03	0.16	0.7 [0;2]	0.9 [0;3]	6 [4;8]	0.1 [0;1.1]	0.1 [0;1.4]
Introduction (mo)—Baby cereals	0.58	0.12	0.25	0.13	−0.45	8 [6;12]	8 [6;12]	9 [7;12]	6 [5;7]	6 [5;8]
Introduction (mo)—Fruit/Vegetables	0.61	−0.14	0.07	0.04	0.01	6 [5;6]	6 [5;7]	6 [5;6]	5 [5;6]	5 [4;6]
Introduction (mo)—Potatoes	0.55	−0.19	−0.18	0.01	−0.02	6 [6;7]	7 [6;8]	6 [6;7]	6 [5;7]	6 [5;7]
Introduction (mo)—Meat/fish/eggs	0.61	−0.16	−0.18	0.08	0.06	7 [7;8]	8 [7;9]	7 [7;8]	7 [6;7]	7 [6;8]
Introduction (mo)—Dairy products	0.61	−0.15	−0.01	−0.03	−0.06	7 [6;8]	8 [7;9]	7 [6;8]	7 [6;7]	6 [6;7]
Introduction (mo)—Bread/pasta/rice	0.50	−0.39	0.08	0.17	0.29	8 [7;9]	10 [8;12]	8 [7;9]	8 [7;9]	8 [7;9]
Introduction (mo)—Sweetened beverages & fruit juices	0.48	−0.26	0.35	0.27	−0.26	12 [12;12]	12 [12;12]	12 [7;12]	7 [5;9]	10 [6;12]
Introduction (mo)—Cow’s milk	0.34	−0.25	0.28	−0.80	−0.01	12 [12;12]	12 [12;12]	12 [12;12]	12 [12;12]	8 [7;10]
Introduction (mo)—Food pieces	0.34	−0.46	0.20	0.14	0.42	9 [8;12]	10 [9;12]	8 [7;10]	9 [7;10]	8 [7;10]
Number of breast feeds at CFI	0.39	0.79	−0.05	−0.04	0.21	0 [0;0]	0 [0;1]	5 [4;7]	0 [0;0]	0 [0;0]
Number of bottle feeds at CFI	−0.46	−0.70	−0.07	−0.02	−0.20	4 [4;4]	4 [3;4]	0 [0;1]	4 [4;5]	4 [4;4]
Frequency * (0–4)—Baby cereals	−0.45	−0.13	−0.12	−0.12	0.52	2 [0;4]	2 [0;4]	2 [0;3]	4 [2;4]	3 [2;4]
Frequency (0–5)—Fruits/vegetables	−0.24	0.12	0.66	0.06	0.12	5 [5;5]	4 [3;5]	5 [4;5]	5 [4;5]	5 [4;5]
Frequency (0–4)—Potatoes	−0.19	0.17	0.63	0.10	0.14	3 [3;3]	2 [2;3]	3 [2;3]	3 [2;3]	3 [2;3]
Frequency (0–4)—Meat/fish/eggs	−0.29	0.11	0.65	0.05	0.06	3 [3;4]	3 [2;4]	3 [3;4]	3 [3;4]	3 [3;4]
Frequency (0–4)—Dairy products	−0.36	0.08	0.41	0.11	0.15	4 [3;4]	2 [2;4]	3 [2;4]	4 [3;4]	4 [3;4]
Frequency (0–4)—Bread/pasta/rice	−0.35	0.36	0.17	−0.14	−0.31	2 [2;3]	2 [0;2]	2 [2;3]	2 [2;3]	2 [2;3]
Frequency (0–4)—Sweetened beverages & fruit juices	−0.44	0.26	−0.34	−0.28	0.27	0 [0;0]	0 [0;0]	0 [0;2]	2 [2;2]	1 [0;2]
Frequency (0–4)—Cow’s milk	−0.32	0.23	−0.27	0.81	0.01	0 [0;0]	0 [0;0]	0 [0;0]	0 [0;0]	2 [2;4]
Frequency (0–3)—Food pieces	−0.28	0.42	−0.09	−0.14	−0.46	1 [0;1]	1 [0;1]	1 [1;2]	1 [1;2]	1 [1;2]

Data are factor loadings for the PCA and median [Q1–Q3] for the HAC. * Introduction: introduced more than once. Frequency: median frequency after introduction. Pattern 1 was labelled “B1—later CF, longer BF”; Pattern 2 “B2—very long BF, higher BF rate, earlier food pieces”; Pattern 3 “B3—frequent intake of main food groups”; Pattern 4 “B4—earlier cow’s milk”; Pattern 5 “B5—earlier baby cereals and later food pieces”. Cluster 1 was labelled “B1′—intermediate BF, intermediate CF, no sweetened beverages and fruit juices”; Cluster 2 “B2′—intermediate BF, late CF, late food pieces, infrequent main food groups”, Cluster 3 “B3′—long BF, intermediate CF, early food pieces”, Cluster 4 “B4′—short BF, early CF, very early sweetened beverages and fruit juices” and Cluster 5 “B5′—short BF, early CF and very early cow’s milk”. BF, breastfeeding; CF, complementary feeding. CFI, complementary food introduction.

**Table 3:** Analysis of core variables (BF and main food groups), food pieces, organic and commercial complementary foods, Set C of variables (*n* = 8.922).

	Principal Component Analysis (PCA)					Hierarchical Ascendant Classification (HAC)				
	Pattern 1	Pattern 2	Pattern 3	Pattern 4	Pattern 5	Cluster 1	Cluster 2	Cluster 3	Cluster 4	Cluster 5
% variance explained (PCA) or % (*n*) (HAC)	18%	14%	9%	7%	6%	32% (2826)	19% (1728)	19% (1715)	18% (1610)	12% (1043)
Any BF duration (mo)	0.54	0.72	−0.04	−0.02	0.14	1.4 [0;3.8]	9.8 [7;14]	1.4 [0;4]	0.7 [0;2.8]	0.7 [0;2.8]
Introduction * (mo)—Infant formula	0.55	0.66	0.00	−0.03	0.14	0.7 [0;2]	6 [4;8]	0.7 [0;2]	0.2 [0;1.3]	0.2 [0;1.4]
Introduction (mo)—Baby cereals	0.58	0.10	0.23	0.14	−0.39	8 [6;12]	9 [7;12]	8 [6;12]	6 [5;7]	6 [5;8]
Introduction (mo)—Fruits/vegetables	0.60	−0.15	0.07	0.03	0.01	6 [5;6]	6 [5;6]	6 [5;6]	5 [5;6]	5 [5;6]
Introduction (mo)—Potatoes	0.54	−0.21	−0.18	0.00	−0.02	6 [6;7]	6 [6;7]	7 [6;8]	6 [5;7]	6 [5;7]
Introduction (mo)—Meat/fish/eggs	0.60	−0.18	−0.17	0.07	0.06	7 [7;8]	7 [7;8]	8 [7;9]	7 [6;7]	7 [6;8]
Introduction (mo)—Dairy products	0.60	−0.16	0.00	−0.05	−0.06	7 [6;8]	7 [6;8]	8 [7;10]	6 [6;7]	6 [6;7]
Introduction (mo)—Bread/pasta/rice	0.49	−0.41	0.09	0.19	0.32	8 [7;9]	8 [7;9]	9 [8;12]	8 [7;9]	8 [7;9]
Introduction (mo)—Sweetened beverages & fruit juice	0.47	−0.27	0.35	0.23	−0.35	12 [12;12]	12 [8;12]	12 [12;12]	7 [5;9]	10 [6;12]
Introduction (mo)—Cow’s milk	0.33	−0.25	0.28	−0.79	0.02	12 [12;12]	12 [12;12]	12 [12;12]	12 [12;12]	8 [7;10]
Introduction (mo)—Food pieces	0.32	−0.47	0.20	0.15	0.40	9 [8;12]	9 [7;10]	10 [9;12]	9 [7;10]	8 [7;10]
Number of breast feeds at CFI	0.41	0.77	−0.08	−0.03	0.20	0 [0;0]	5 [4;7]	0 [0;0]	0 [0;0]	0 [0;0]
Number of bottle feeds at CFI	−0.48	−0.68	−0.04	−0.03	−0.18	4 [4;4]	0 [0;1]	4 [4;4]	4 [4;5]	4 [4;4]
Frequency * (0–4)—Baby cereals	−0.45	−0.10	−0.10	−0.13	0.46	2 [0;4]	2 [0;3]	2 [0;4]	4 [2;4]	3 [2;4]
Frequency (0–5)—Fruit/vegetables	−0.23	0.15	0.66	0.06	0.12	5 [5;5]	5 [4;5]	4 [3;5]	5 [4;5]	5 [4;5]
Frequency (0–4)—Potatoes	−0.17	0.19	0.63	0.12	0.15	3 [3;3]	3 [2;4]	2 [2;3]	3 [2;3]	3 [2;3]
Frequency (0–4)—Meat/fish/eggs	−0.27	0.14	0.66	0.07	0.07	3 [3;4]	3 [3;4]	3 [2;3]	3 [3;4]	3 [3;4]
Frequency (0–4)—Dairy products	−0.35	0.09	0.40	0.13	0.15	4 [3;4]	3 [2;4]	2 [2;4]	4 [3;4]	4 [3;4]
Frequency (0–4)—Bread/pasta/rice	−0.34	0.37	0.17	−0.16	−0.34	2 [2;3]	2 [2;3]	2 [0;2]	2 [2;3]	2 [2;3]
Frequency (0–4)—Sweetened beverages & fruit juices	−0.43	0.27	−0.33	−0.24	0.35	0 [0;0]	0 [0;2]	0 [0;0]	2 [2;2]	1 [0;2]
Frequency (0–4)—Cow’s milk	−0.31	0.24	−0.27	0.80	−0.02	0 [0;0]	0 [0;0]	0 [0;0]	0 [0;0]	2 [2;3]
Frequency (0–3)—Food pieces	−0.27	0.44	−0.09	−0.15	−0.44	1 [0;1]	1 [1;2]	1 [0;1]	1 [1;2]	1 [1;2]
Frequency (0–3)—Organic foods	0.18	0.29	0.16	−0.06	0.00	1 [0;2]	1 [0;2]	0 [0;1]	1 [0;2]	0 [0;1]
Frequency (0–3)—Commercial complementary foods	−0.23	0.00	−0.03	−0.16	−0.16	1 [1;2]	1 [1;2]	1 [0;2]	2 [1;2]	1 [1;2]

Values are factor loadings for the PCA and median [Q1–Q3] for the HAC. * Introduction: introduced more than once. Frequency: median frequency after introduction. Pattern 1 was labelled “C1—later CF, longer BF”; Pattern 2 “C2—very longer BF, higher BF rate, earlier food pieces”; Pattern 3 “C3—frequent intake of main food groups”; Pattern 4 “C4—earlier cow’s milk”; Pattern 5 “C5—frequent baby cereals, later food pieces”. Cluster 1 was Labelled “C1′—intermediate BF, intermediate CF”; Cluster 2 “C2′—long BF, intermediate CF”, Cluster 3 “C3′—intermediate BF, late CF”, Cluster 4 “C4′—short BF, early CF, very early sweetened beverages and fruit juices, frequent commercial complementary foods” and Cluster 5 “C5′—short BF, early CF and very early cow’s milk”. CFI, complementary food introduction.

**Table 4:** Multivariable linear regression analysis explaining each pattern (identified by principal component analysis (PCA)) by family characteristics (*n* = 7.556).

	B1—Later CF, Longer BF	*p* Value	B2—Very Long BF, Higher BF Rate, Earlier Food Pieces	*p* Value	B3—Frequent Intake of Main Food Groups	*p* Value	B4—Earlier Cow’s Milk	*p* Value	B5—Earlier Baby Cereals, Later Food Pieces	*p* Value
Maternal age (years)		<0.001		0.02		<0.001		0.63		0.05
<25	−0.6 [−0.7;−0.5]		0.1 [0.0;0.3]		−0.2 [−0.3;−0.1]		0.0 [−0.1;0.1]		0.0 [−0.1;0.1]	
25–29	−0.2 [−0.2;−0.1]		0.1 [0.0;0.1]		0.0 [0.0;0.1]		0.0 [−0.1;0.0]		0.1 [0.0;0.1]	
30–34	0 [Ref]		0 [Ref]		0 [Ref]		0 [Ref]		0 [Ref]	
≥35	0.0 [0.0;0.1]		0 [0.0;0.1]		−0.1 [−0.2;−0.1]		0.0 [−0.1;0.0]		0.0 [0.0;0.1]	
Maternal education level		<0.001		<0.001		<0.001		0.04		0.88
Up to lower secondary	−0.4 [−0.6;−0.3]		−0.3 [−0.4;−0.1]		−0.5 [−0.6;−0.3]		−0.1 [−0.3;0.1]		0.0 [−0.1;0.2]	
Upper secondary	−0.5 [−0.6;−0.4]		−0.3 [−0.3;−0.2]		−0.3 [−0.4;−0.2]		0.0 [−0.1;0.1]		0.0 [−0.1;0.0]	
Intermediate	−0.3 [−0.4;−0.2]		−0.2 [−0.2;−0.1]		−0.1 [−0.2;0.0]		−0.1 [−0.1;0.0]		0.0 [−0.1;0.0]	
3-year university degree	−0.1 [−0.1;0.0]		−0.1 [−0.1;0.0]		0.0 [0.0;0.1]		0.0 [0.0;0.1]		0.0 [−0.1;0.1]	
At least 5-year university degree	0 [Ref]		0 [Ref]		0 [Ref]		0 [Ref]		0 [Ref]	
Family income (€/month/consumption unit)		0.19		<0.001		<0.001		0.34		0.07
≤750	0.0 [−0.2;0.1]		0.3 [0.2;0.4]		−0.4 [−0.6;−0.3]		0.1 [−0.1;0.2]		−0.1 [−0.2;0.0]	
751–1111	0.0 [−0.1;0.1]		0.2 [0.1;0.3]		−0.2 [−0.3;−0.2]		0.0 [−0.1;0.1]		0.0 [0.0;0.1]	
1112–1500	0.0 [0.0;0.1]		0.1 [0.0;0.1]		−0.1 [−0.1;0.0]		0.0 [−0.1;0.0]		0.0 [−0.1;0.1]	
1501–1944	0 [Ref]		0 [Ref]		0 [Ref]		0 [Ref]		0 [Ref]	
1945–2500	0.1 [0.0;0.1]		−0.1 [−0.1;0.0]		0.0 [−0.1;0.1]		0.0 [0.0;0.1]		0.0 [−0.1;0.0]	
>2500	0.0 [−0.1;0.1]		−0.1 [−0.2;0.0]		0.1 [0.0;0.1]		0.1 [0.0;0.1]		−0.1 [−0.2;0.0]	
Migration status		0.03		<0.001		<0.001		<0.001		0.54
Migrant	−0.1 [−0.2;0.0]		0.6 [0.5;0.6]		−0.2 [−0.3;−0.1]		−0.3 [−0.4;−0.2]		0.0 [−0.1;0.1]	
Descendant of migrant	−0.1 [−0.2;0.0]		0.2 [0.1;0.2]		−0.1 [−0.1;0.0]		−0.1 [−0.2;0.0]		0.0 [−0.1;0.0]	
Majority of population	0 [Ref]		0 [Ref]		0 [Ref]		0 [Ref]		0 [Ref]	
Single motherhood		0.07		0.50		<0.001		0.003		0.29
No	0 [Ref]		0 [Ref]		0 [Ref]		0 [Ref]		0 [Ref]	
Yes	−0.1 [−0.3;0.0]		0.1 [−0.1;0.2]		−0.3 [−0.5;−0.1]		−0.2 [−0.4;−0.1]		−0.1 [−0.3;0.1]	
Older children in the household		<0.001		<0.001		<0.001		<0.001		<0.001
No older child	0 [Ref]		0 [Ref]		0 [Ref]		0 [Ref]		0 [Ref]	
One older child	0.1 [0.1;0.2]		0.0 [−0.1;0.0]		−0.1 [−0.2;−0.1]		0.1 [0.1;0.2]		−0.1 [−0.2;−0.1]	
At least 2 older children	0.1 [0.0;0.1]		0.2 [0.1;0.2]		−0.2 [−0.3;−0.1]		0.2 [0.1;0.3]		−0.1 [−0.2;0.0]	
Child’s sex		<0.001		0.06		0.35		0.15		0.008
Boys	−0.1 [−0.2;−0.1]		0 [0.0;0.1]		0.0 [0.0;0.1]		0.0 [−0.1;0.0]		0.1 [0.0;0.1]	
Girls	0 [Ref]		0 [Ref]		0 [Ref]		0 [Ref]		0 [Ref]	
Child’s age at maternal return to work		<0.001		<10^−5^		0.01		0.61		<0.001
Not concerned	0.1 [0.1;0.2]		0.5 [0.4;0.5]		−0.1 [−0.2;0.0]		0.0 [−0.1;0.1]		0.1 [0.0;0.2]	
<10 weeks	0.0 [−0.1;0.0]		0.0 [0.0;0.1]		0.0 [−0.1;0.0]		0.0 [−0.1;0.0]		0.0 [−0.1;0.1]	
10–13 weeks	0 [Ref]		0 [Ref]		0 [Ref]		0 [Ref]		0 [Ref]	
3 to <6 months	0.1 [0.1;0.2]		0.1 [0.1;0.2]		0.0 [0.0;0.1]		0.0 [−0.1;0.0]		0.0 [−0.1;0.1]	
After 6 months	0.3 [0.2;0.3]		0.5 [0.4;0.5]		0.0 [−0.1;0.1]		0.0 [−0.1;0.1]		0.2 [0.1;0.2]	

Values are adjusted β [95% CI] also adjusted for variables related to study design (region, recruitment wave and maternity size). “B1—later CF, longer BF”; Pattern 2 “B2—very long BF, higher BF rate, earlier food pieces”; Pattern 3 “B3—frequent intake of main food groups”; Pattern 4 “B4—earlier cow’s milk”; Pattern 5 “B5—earlier baby cereals and later food pieces”. Cluster 1 was labelled “B1′—intermediate BF, intermediate CF, no sweetened beverages and fruit juices”; Cluster 2 “B2′—intermediate BF, late CF, late food pieces, infrequent main food groups”, Cluster 3 “B3′—long BF, intermediate CF, early food pieces”, Cluster 4 “B4′—short BF, early CF, very early sweetened beverages and fruit juices” and Cluster 5 “B5′. BF: breast feeding; CF: complementary feeding.

**Table 5:** Multivariable linear regression analysis explaining each pattern (identified by principal component analysis (PCA)) by family characteristics (not shown) and health-related variables (*n* = 7.556).

	B1—Later CF, Longer BF	*p* Value	B2—Very Long BF, Higher BF Rate, Earlier Food Pieces	*p* Value	B3—Frequent Intake of Main Food Groups	*p* Value	B4—Earlier Cow’s Milk	*p* Value	B5—Earlier Baby Cereals, Later Food Pieces	*p* Value
Smoking during pregnancy		<0.001		0.05		0.30		0.60		0.70
Never smoker	0 [Ref]		0 [Ref]		0 [Ref]		0 [Ref]		0 [Ref]	
Smoker only before pregnancy	−0.1 [−0.1;0.0]		0.0 [−0.1;0.0]		0.0 [0.0;0.1]		0.0 [−0.1;0.0]		0.0 [−0.1;0.0]	
Smoker only in early pregnancy	−0.1 [−0.2;0.0]		0.0 [−0.1;0.1]		0.1 [−0.1;0.2]		−0.1 [−0.2;0.1]		−0.1 [−0.2;0.1]	
Smoker throughout pregnancy	−0.3 [−0.4;−0.2]		−0.1 [−0.2;0.0]		0.0 [−0.1;0.1]		0.0 [−0.1;0.1]		0.0 [−0.1;0.1]	
Pre-pregnancy BMI (kg/m2)		<0.001		0.40		0.008		0.30		0.60
<18.5	0.1 [0.0;0.2]		0.0 [−0.1;0.0]		0.0 [−0.1;0.1]		0.0 [0.0;0.1]		0.0 [−0.1;0.1]	
18.5–24.9	0 [Ref]		0 [Ref]		0 [Ref]		0 [Ref]		0 [Ref]	
25–29.9	−0.1 [−0.2;−0.1]		0.0 [−0.1;0.0]		−0.1 [−0.2;0.0]		0.1 [0.0;0.1]		0.0 [−0.1;0.1]	
≥30	−0.2 [−0.3;−0.1]		0.0 [−0.1;0.0]		−0.1 [−0.2;0.0]		0.0 [−0.1;0.1]		−0.1 [−0.1;0.0]	
Physician consulted after hospital discharge		0.80		0.20		<0.001		0.10		0.80
Paediatrician	0.0 [−0.1;0.0]		0.0 [−0.1;0.0]		0.1 [0.1;0.2]		−0.1 [−0.1;0.0]		0.0 [0.0;0.1]	
Other child doctor	0.0 [−0.1;0.1]		0.0 [0.0;0.1]		0.1 [0.0;0.1]		0.0 [−0.1;0.1]		0.0 [−0.1;0.1]	
General practitioner	0 [Ref]		0 [Ref]		0 [Ref]		0 [Ref]		0 [Ref]	
None/Other	0.0 [−0.1;0.1]		0.0 [−0.1;0.1]		−0.1 [−0.2;0.0]		0.0 [−0.1;0.1]		0.0 [−0.1;0.1]	
Attendance at pre-birth preparation sessions		<0.001		<0.001		<0.001		0.60		0.10
None	−0.2 [−0.3;−0.2]		−0.2 [−0.2;−0.1]		−0.1 [−0.2;−0.1]		0.0 [−0.1;0.0]		−0.1 [−0.1;0.0]	
<7	−0.1 [−0.1;0.0]		0.0 [−0.1;0.0]		0.0 [−0.1;0.0]		0.0 [−0.1;0.1]		0.0 [−0.1;0.0]	
≥7	0 [Ref]		0 [Ref]		0 [Ref]		0 [Ref]		0 [Ref]	

“B1—later CF, longer BF”; Pattern 2 “B2—very long BF, higher BF rate, earlier food pieces”; Pattern 3 “B3—frequent intake of main food groups”; Pattern 4 “B4—earlier cow’s milk”; Pattern 5 “B5—earlier baby cereals and later food pieces”. Cluster 1 was labelled “B1′—intermediate BF, intermediate CF, no sweetened beverages and fruit juices”; Cluster 2 “B2′—intermediate BF, late CF, late food pieces, infrequent main food groups”, Cluster 3 “B3′—long BF, intermediate CF, early food pieces”, Cluster 4 “B4′—short BF, early CF, very early sweetened beverages and fruit juices” and Cluster 5 “B5′. BF: breast feeding; CF: complementary feeding.

**Table 6:** Multivariable multinomial logistic regression analysis explaining clusters (identified by HAC) by family characteristics (*n* = 7.556).

	B2′—Intermediate BF, Late CF, Late Food Pieces, Infrequent of Main Food Groups	B3′—Long BF, Intermediate CF, Early Food Pieces	B4′—Short BF, Early CF, Very Early Sweetened Beverages & Fruit Juices	B5′—Short BF, Early CF, Very Early Cow’s Milk	*p* Value
Maternal age (years)					<0.001
<25	0.8 [0.5–1.1]	1.0 [0.7–1.5]	2.2 [1.6–2.9]	2.1 [1.5–3.0]	
25–29	0.8 [0.7–1.0]	1.1 [1.0–1.4]	1.3 [1.1–1.5]	1.2 [1.0–1.5]	
30–34	1 [ref]	1 [Ref]	1 [Ref]	1 [Ref]	
≥35	1.3 [1.1–1.6]	1.3 [1.1–1.5]	1.4 [1.1–1.7]	1.2 [0.9–1.4]	
Maternal education level					<0.001
Up to lower secondary	1.2 [0.7–2.1]	0.6 [0.3–1.0]	2.5 [1.5–4.2]	1.5 [0.8–2.9]	
Upper secondary	1.2 [1.0–1.5]	0.5 [0.4–0.6]	2.1 [1.7–2.7]	2.0 [1.5–2.6]	
Intermediate	1.0 [0.8–1.2]	0.7 [0.5–0.8]	1.7 [1.4–2.1]	1.3 [1.0–1.6]	
3-year university degree	0.8 [0.6–1.0]	0.8 [0.6–0.9]	0.9 [0.7–1.2]	1.0 [0.7–1.3]	
At least 5-year university degree	1 [ref]	1 [Ref]	1 [Ref]	1 [Ref]	
Family income (€/month/consumption unit)					<0.001
≤750	1.4 [0.9–2.2]	2.7 [1.7–4.1]	1.7 [1.1–2.6]	2.0 [1.3–3.3]	
751–1111	1.1 [0.8–1.4]	1.8 [1.3–2.4]	1.1 [0.9–1.5]	1.6 [1.1–2.2]	
1112–1500	1.0 [0.9–1.2]	1.3 [1.1–1.6]	1.0 [0.8–1.2]	1.1 [0.9–1.4]	
1501–1944	1 [ref]	1 [Ref]	1 [Ref]	1 [Ref]	
1945–2500	1.0 [0.9–1.3]	0.9 [0.7–1.1]	0.8 [0.6–1.0]	0.8 [0.6–1.1]	
>2500	0.8 [0.6–1.0]	0.7 [0.6–1.0]	0.8 [0.6–1.0]	0.8 [0.6–1.1]	
Migration status					<0.001
Migrant	1.0 [0.7–1.4]	2.7 [2–3.5]	1.8 [1.3–2.5]	0.9 [0.6–1.4]	
Descendant of migrant	1.0 [0.8–1.3]	1.3 [1–1.6]	1.3 [1.0–1.6]	1.1 [0.8–1.5]	
Majority of population	1 [ref]	1 [Ref]	1 [Ref]	1 [Ref]	
Single motherhood					0.12
No	1 [ref]	1 [Ref]	1 [Ref]	1 [Ref]	
Yes	1.0 [0.6–1.8]	1.1 [0.6–2.0]	1.8 [1.1–2.9]	1.3 [0.7–2.4]	
Older children in the household					<0.001
No older child	1 [ref]	1 [Ref]	1 [Ref]	1 [Ref]	
One older child	1.2 [1.0–1.4]	1.1 [0.9–1.3]	0.7 [0.6–0.8]	1.1 [0.9–1.3]	
At least 2 older children	1.1 [0.9–1.4]	1.6 [1.2–2.0]	0.8 [0.6–1.0]	1.9 [1.4–2.5]	
Child’s sex					<0.001
Boys	1.0 [0.9–1.1]	1.1 [0.9–1.2]	1.3 [1.1–1.5]	1.2 [1.0–1.4]	
Girls	1 [ref]	1 [Ref]	1 [Ref]	1 [Ref]	
Child’s age at maternal return to work					<0.001
Not concerned	1.2 [0.9–1.5]	3.9 [3.1–5.0]	1.3 [1.0–1.7]	1.4 [1.1–1.9]	
<10 weeks	0.8 [0.7–1.0]	1.1 [0.8–1.4]	1.1 [0.9–1.4]	1.0 [0.8–1.3]	
10–13 weeks	1 [Ref]	1 [Ref]	1 [Ref]	1 [Ref]	
3 to <6 months	1.0 [0.9–1.3]	1.8 [1.5–2.3]	0.9 [0.7–1.1]	0.9 [0.7–1.1]	
After 6 months	1.3 [1.0–1.5]	4.3 [3.5–5.4]	1.2 [0.9–1.4]	1.2 [0.9–1.6]	

Reference group: B1′—intermediate BF and intermediate CF. Values are odds ratios (OR) [95% confidence intervals [CI]] adjusted on study design variables (region, recruitment wave and maternity size). Pattern 2 “B2—very long BF, higher BF rate, earlier food pieces”; Pattern 3 “B3—frequent intake of main food groups”; Pattern 4 “B4—earlier cow’s milk”; Pattern 5 “B5—earlier baby cereals and later food pieces”. Cluster 1 was labelled “B1′—intermediate BF, intermediate CF, no sweetened beverages and fruit juices”; Cluster 2 “B2′—intermediate BF, late CF, late food pieces, infrequent main food groups”, Cluster 3 “B3′—long BF, intermediate CF, early food pieces”, Cluster 4 “B4′—short BF, early CF, very early sweetened beverages and fruit juices” and Cluster 5 “B5′. BF: breast feeding; CF: complementary feeding.

**Table 7:** Multivariable multinomial logistic regression analysis explaining clusters (identified by HAC) by family characteristics (not shown) and health-related variables (*n* = 7.556).

	B2′—Intermediate BF, Late CF, Late Food Pieces, Infrequent of Main Food Groups	B3′—Long BF, Intermediate CF, Early Food Pieces	B4′—Short BF, Early CF, Very Early Sweetened Beverages & Fruit Juices	B5′—Short BF, Early CF, Very Early Cow Milk	*p* Value
Smoking during pregnancy					<0.001
Never smoker	1 [Ref]	1 [Ref]	1 [Ref]	1 [Ref]	
Smoker only before pregnancy	0.8 [0.7–0.9]	0.8 [0.7–0.9]	1.0 [0.8–1.2]	0.9 [0.8–1.2]	
Smoker only in early pregnancy	0.7 [0.5–1.1]	0.9 [0.6–1.3]	0.9 [0.6–1.3]	1.0 [0.7–1.6]	
Smoker throughout pregnancy	0.7 [0.6–0.9]	0.6 [0.4–0.7]	1.2 [0.9–1.5]	1.3 [1.0–1.7]	
Pre-pregnancy BMI (kg/m^2^)					0.002
<18.5	1.2 [1.0–1.6]	1.1 [0.9-1.9]	0.9 [0.7-1.3]	1.2 [0.9-1.7]	
18.5–24.9	1 [Ref]	1 [Ref]	1 [Ref]	1 [Ref]	
25–29.9	1.2 [1.0-1.5]	1.0 [0.8-1.2]	1.2 [1.0-1.5]	1.5 [1.2-1.9]	
≥30	1.0 [0.8-1.3]	0.8 [0.6-1.1]	1.2 [0.9-1.5]	1.3 [1.0-1.7]	
Physician consulted after hospital discharge					0.002
Paediatrician	0.9 [0.7–1.0]	0.8 [0.7–1.0]	0.9 [0.7–1.0]	0.7 [0.6–0.8]	
Other child doctor	0.9 [0.7–1.1]	1.1 [0.9–1.4]	0.9 [0.7–1.1]	0.8 [0.6–1.1]	
General practitioner	1 [Ref]	1 [Ref]	1 [Ref]	1 [Ref]	
None/other	1.0 [0.7–1.4]	1.2 [0.9–1.7]	1.2 [0.8–1.5]	1.2 [0.8–1.7]	
Attendance at pre-birth preparation sessions					<0.001
None	1.1 [0.9–1.3]	0.6 [0.5–0.7]	1.3 [1.1–1.6]	1.2 [1.0–1.6]	
<7	1.0 [0.9–1.2]	0.9 [0.7–1.0]	1.0 [0.9–1.2]	1.1 [0.9–1.3]	
≥7	1 [Ref]	1 [Ref]	1 [Ref]	1 [Ref]	

Reference group: B1′—intermediate BF and intermediate CF. Values are OR [95% CI] adjusted on familial characteristics (Maternal age, maternal education level, family monthly income per consumption unit, migration status, single motherhood, older children in the household, child’s age at maternal return to work, child’s sex) and variables related to study design (region, recruitment wave and maternity size). Pattern 2 “B2—very long BF, higher BF rate, earlier food pieces”; Pattern 3 “B3—frequent intake of main food groups”; Pattern 4 “B4—earlier cow’s milk”; Pattern 5 “B5—earlier baby cereals and later food pieces”. Cluster 1 was labelled “B1′—intermediate BF, intermediate CF, no sweetened beverages and fruit juices”; Cluster 2 “B2′—intermediate BF, late CF, late food pieces, infrequent main food groups”, Cluster 3 “B3′—long BF, intermediate CF, early food pieces”, Cluster 4 “B4′—short BF, early CF, very early sweetened beverages and fruit juices” and Cluster 5 “B5′. BF: breast feeding; CF: complementary feeding.

## Data Availability

Data from French Nationwide ELFE.

## Supplementary Materials

The following are available online at https://www.mdpi.com/2072-6643/13/1/33/s1, Table S1: sensitivity analysis with multiple imputations: multivariable linear regression analysis explaining each pattern (identified by PCA) by family characteristics. Table S2: sensitivity analysis with weighted data: multivariable linear regression analysis explaining each pattern (identified by PCA) by family characteristics. Table S3: sensitivity analysis with multiple imputations: multivariable multinomial logistic regression analysis explaining clusters (identified by HAC) by family characteristics all together. Table S4: sensitivity analysis with weighted data: multivariable multinomial logistic regression explaining clusters (identified by HAC) by family characteristics all together.
