# Supplementary material for: Characterization of Infant Feeding Practices and Related-Family Characteristics in the French Nationwide ELFE Birth Cohort
Source: Nutrients. 2020 Dec 24;13(1):33. doi: 10.3390/nu13010033 (PMC7824677; doi:10.3390/nu13010033)
Supplement: Supplementary file 1 [file nutrients-13-00033-s001.pdf]

Supplementary Table 1. Sensitivity analysis with multiple imputations: multivariable linear regression analysis explaining each pattern (identified by PCA) by family characteristics (N=8.922).

	B1- late CF, long BF	B2- very long BF, high BF rate, early unmixed foods	B3- frequent intake of main food groups	B4- early cow's milk	B5- early baby cereals, late unmixed foods
Maternal age (years)					
< 25	-0.55 [-0.65 ; -0.46]	0.19 [0.09 ; 0.28]	-0.20 [-0.30 ; -0.11]	0.02 [-0.08 ; 0.12]	0.00 [-0.10 ; 0.10]
25–29	-0.16 [-0.21 ; -0.11]	0.05 [0.00 ; 0.10]	-0.01 [-0.06 ; 0.04]	-0.02 [-0.07 ; 0.03]	0.06 [0.01 ; 0.11]
30–34	0 [Ref]	0 [Ref]	0 [Ref]	0 [Ref]	0 [Ref]
≥ 35	0.06 [0.01 ; 0.11]	0.01 [-0.04 ; 0.06]	-0.15 [-0.2 ; -0.09]	-0.05 [-0.11 ; 0.00]	0.00 [-0.05 ; 0.06]
Maternal education level					
Up to lower secondary	-0.45 [-0.61 ; -0.29]	-0.29 [-0.44 ; -0.13]	-0.46 [-0.62 ; -0.31]	-0.04 [-0.21 ; 0.13]	-0.01 [-0.16 ; 0.15]
Upper secondary	-0.49 [-0.55 ; -0.42]	-0.26 [-0.32 ; -0.19]	-0.31 [-0.37 ; -0.24]	-0.02 [-0.09 ; 0.05]	-0.01 [-0.08 ; 0.06]
Intermediate	-0.28 [-0.34 ; -0.22]	-0.16 [-0.22 ; -0.10]	-0.13 [-0.19 ; -0.07]	-0.08 [-0.15 ; -0.02]	-0.01 [-0.08 ; 0.05]
3-year university degree	-0.05 [-0.12 ; 0.01]	-0.05 [-0.11 ; 0.01]	-0.01 [-0.07 ; 0.05]	0.00 [-0.07 ; 0.06]	0.02 [-0.05 ; 0.08]
At least 5-year university degree	0 [Ref]	0 [Ref]	0 [Ref]	0 [Ref]	0 [Ref]
Family income (€/month/consumption unit)					
≤750	-0.08 [-0.20 ; 0.04]	0.30 [0.19 ; 0.42]	-0.4 [-0.51 ; -0.28]	0.07 [-0.05 ; 0.19]	-0.09 [-0.21 ; 0.03]
751–1111	0.00 [-0.08 ; 0.08]	0.16 [0.08 ; 0.24]	-0.21 [-0.30 ; -0.13]	0.00 [-0.09 ; 0.08]	0.03 [-0.05 ; 0.12]
1112–1500	0.01 [-0.05 ; 0.07]	0.08 [0.02 ; 0.13]	-0.07 [-0.12 ; -0.01]	-0.03 [-0.09 ; 0.03]	0.00 [-0.06 ; 0.06]
1501–1944	0 [Ref]	0 [Ref]	0 [Ref]	0 [Ref]	0 [Ref]
1945–2500	0.07 [0.01 ; 0.14]	-0.05 [-0.11 ; 0.01]	-0.01 [-0.07 ; 0.06]	0.02 [-0.05 ; 0.09]	-0.04 [-0.10 ; 0.03]
>2500	0.00 [-0.08 ; 0.08]	-0.11 [-0.18 ; -0.03]	0.06 [-0.02 ; 0.13]	0.04 [-0.04 ; 0.13]	-0.11 [-0.19 ; -0.03]
Migration status					
Migrant	-0.07 [-0.16 ; 0.01]	0.58 [0.50 ; 0.66]	-0.22 [-0.30 ; -0.13]	-0.27 [-0.36 ; -0.19]	0.00 [-0.09 ; 0.09]
Descendant of migrant	-0.07 [-0.15 ; 0.00]	0.17 [0.10 ; 0.24]	-0.08 [-0.16 ; -0.01]	-0.09 [-0.17 ; -0.02]	-0.04 [-0.11 ; 0.04]
Majority of population	0 [Ref]	0 [Ref]	0 [Ref]	0 [Ref]	0 [Ref]
Single motherhood					
No	0 [Ref]	0 [Ref]	0 [Ref]	0 [Ref]	0 [Ref]
Yes	-0.17 [-0.31 ; -0.03]	0.13 [-0.01 ; 0.28]	-0.28 [-0.43 ; -0.14]	-0.21 [-0.36 ; -0.06]	-0.11 [-0.26 ; 0.04]
Older children in the household					
No older child	0 [Ref]	0 [Ref]	0 [Ref]	0 [Ref]	0 [Ref]
One older child	0.11 [0.06 ; 0.15]	-0.01 [-0.06 ; 0.04]	-0.11 [-0.16 ; -0.06]	0.11 [0.06 ; 0.16]	-0.11 [-0.16 ; -0.06]
At least 2 older children	0.08 [0.01 ; 0.15]	0.15 [0.08 ; 0.22]	-0.20 [-0.27 ; -0.13]	0.19 [0.12 ; 0.26]	-0.08 [-0.15 ; -0.01]
Child's sex					
Boys	0 [Ref]	0 [Ref]	0 [Ref]	0 [Ref]	0 [Ref]
Girls	0.11 [0.08 ; 0.15]	-0.05 [-0.09 ; -0.01]	-0.02 [-0.06 ; 0.02]	0.01 [-0.03 ; 0.05]	-0.04 [-0.08 ; 0.00]
Child's age at maternal return to work					
Not concerned	0.11 [0.04 ; 0.19]	0.43 [0.35 ; 0.50]	-0.15 [-0.22 ; -0.08]	-0.01 [-0.08 ; 0.07]	0.10 [0.02 ; 0.17]
<10 weeks	-0.03 [-0.10 ; 0.04]	0.05 [-0.02 ; 0.11]	-0.04 [-0.10 ; 0.03]	-0.05 [-0.11 ; 0.02]	-0.03 [-0.10 ; 0.04]
10–13 weeks	0 [Ref]	0 [Ref]	0 [Ref]	0 [Ref]	0 [Ref]
3 to <6 months	0.11 [0.05 ; 0.17]	0.14 [0.08 ; 0.20]	0.02 [-0.04 ; 0.08]	-0.01 [-0.07 ; 0.06]	0.00 [-0.07 ; 0.07]
After 6 months	0.22 [0.16 ; 0.29]	0.42 [0.36 ; 0.49]	-0.01 [-0.07 ; 0.05]	0.03 [-0.04 ; 0.10]	0.17 [0.11 ; 0.24]

Values are adjusted β [95% CI] also adjusted for variables related to study design (region, recruitment wave and maternity size).
BF, breastfeeding; CF, complementary feeding

Supplementary Table 2. Sensitivity analysis with weighted data: multivariable linear regression analysis explaining each pattern (identified by PCA) by family characteristics (N=7.543).

	B1- late CF, long BF	P value	B2- very long BF, high BF rate, early unmixed foods	P value	B3- frequent intake of main food groups	P value	B4- early cow's milk	P value	B5- early baby cereals, late unmixed foods	P value
Maternal age (years)		<0.001		0.02		<0.001		<0.001		<0.001
< 25	-0.6 [-0.7;-0.5]		0.1 [0.0;0.2]		-0.2 [-0.3;-0.1]		0.2 [0.1;0.3]		0.0 [-0.1;0.0]	
25–29	-0.2 [-0.2;-0.1]		0.1 [0.0;0.1]		0.0 [-0.1;0.1]		0.0 [-0.1;0.1]		0.1 [0.0;0.2]	
30–34	0 [Ref]		0 [Ref]		0 [Ref]		0 [Ref]		0 [Ref]	
≥ 35	0 [0;0.1]		0.0 [0.0;0.1]		-0.1 [-0.2;0.0]		0.0 [-0.1;0.0]		0.0 [0.0;0.1]	
Maternal education level		<0.001		<0.001		<0.001		0.001		0.55
Up to lower secondary	-0.3 [-0.4;-0.2]		-0.4 [-0.6;-0.3]		-0.4 [-0.5;-0.2]		-0.2 [-0.3;0.0]		0.0 [-0.2;0.1]	
Upper secondary	-0.6 [-0.6;-0.5]		-0.3 [-0.3;-0.2]		-0.3 [-0.4;-0.2]		0.0 [0.0;0.1]		-0.1 [-0.1;0.0]	
Intermediate	-0.3 [-0.4;-0.2]		-0.1 [-0.2;-0.1]		-0.1 [-0.2;0.0]		-0.1 [-0.1;0.0]		-0.1 [-0.1;0.0]	
3-year university degree	-0.1 [-0.1;0.0]		0.0 [-0.1;0.0]		0.0 [-0.1;0.1]		0.0 [0.0;0.1]		0.0 [-0.1;0.1]	
At least 5-year university degree	0 [Ref]		0 [Ref]		0 [Ref]		0 [Ref]		0 [Ref]	
Family income (€/month/consumption unit)		0.14		<0.001		<0.001		0.45		0.002
≤750	0.0 [-0.1;0.1]		0.3 [0.2;0.4]		-0.4 [-0.6;-0.3]		0.0 [-0.1;0.1]		-0.2 [-0.3;-0.1]	
751–1111	0.1 [0.0;0.2]		0.2 [0.2;0.3]		-0.3 [-0.3;-0.2]		0.0 [-0.1;0.1]		0.0 [-0.1;0.1]	
1112–1500	0.0 [0.0;0.1]		0.1 [0.0;0.2]		-0.1 [-0.1;0.0]		0.0 [-0.1;0.0]		-0.1 [-0.1;0.0]	
1501–1944	0 [Ref]		0 [Ref]		0 [Ref]		0 [Ref]		0 [Ref]	
1945–2500	0.1 [0.0;0.2]		0.0 [-0.1;0.0]		0.0 [-0.1;0.0]		0.0 [-0.1;0.1]		-0.1 [-0.2;0.0]	
>2500	0.0 [-0.1;0.1]		-0.1 [-0.2;0.0]		0.1 [0.0;0.2]		0.0 [-0.1;0.1]		-0.1 [-0.2;0.0]	
Migration status		0.15		<0.001		<0.001		<0.001		0.80
Migrant	0.0 [-0.1;0.0]		0.6 [0.5;0.7]		-0.2 [-0.3;-0.1]		-0.3 [-0.4;-0.2]		0.0 [-0.1;0.1]	
Descendant of migrant	-0.1 [-0.2;0.0]		0.2 [0.1;0.3]		-0.1 [-0.2;0.0]		-0.1 [-0.2;0.0]		0.0 [-0.1;0.1]	
Majority of population	0 [Ref]		0 [Ref]		0 [Ref]		0 [Ref]		0 [Ref]	
Single motherhood		0.02		0.85		0.009		<0.001		0.04
No	0 [Ref]		0 [Ref]		0 [Ref]		0 [Ref]		0 [Ref]	
Yes	-0.2 [-0.3;0.0]		0.0 [-0.1;0.1]		-0.2 [-0.3;0.0]		-0.3 [-0.4;-0.2]		-0.1 [-0.3;0.0]	
Older children in the household		<0.001		<0.001		<0.001		<0.001		<0.001
No older child	0 [Ref]		0 [Ref]		0 [Ref]		0 [Ref]		0 [Ref]	
One older child	0.1 [0.1;0.2]		0.0 [-0.1;0.0]		-0.2 [-0.2;-0.1]		0.1 [0.1;0.2]		-0.1 [-0.2;-0.1]	
At least 2 older children	0.1 [0.0;0.1]		0.2 [0.1;0.2]		-0.2 [-0.3;-0.2]		0.2 [0.2;0.3]		-0.1 [-0.2;0.0]	
Child's sex		<0.001		<0.001		0.51		0.33		0.05
Boys	-0.1 [-0.2;-0.1]		0.1 [0.0;0.1]		0.0 [0.0;0.1]		0.0 [-0.1;0.0]		0.0 [0.0;0.1]	
Girls	0 [Ref]		0 [Ref]		0 [Ref]		0 [Ref]		0 [Ref]	
Child's age at maternal return to work		<0.001		<0.001		<0.001		0.45		<0.001
Not concerned	0.0 [0.0;0.1]		0.4 [0.3;0.5]		-0.2 [-0.2;-0.1]		0.0 [-0.1;0.0]		0.1 [0.0;0.2]	
<10 weeks	-0.1 [-0.1;0.0]		0.1 [0.0;0.1]		0.0 [-0.1;0.1]		-0.1 [-0.1;0.0]		0.0 [-0.1;0.1]	
10–13 weeks	0 [Ref]		0 [Ref]		0 [Ref]		0 [Ref]		0 [Ref]	
3 to <6 months	0.1 [0.0;0.2]		0.1 [0.0;0.2]		0.0 [0.0;0.1]		0.0 [-0.1;0.0]		0.0 [-0.1;0.1]	
After 6 months	0.2 [0.1;0.3]		0.4 [0.3;0.4]		0.0 [-0.1;0.0]		0.0 [-0.1;0.1]		0.1 [0.1;0.2]	

Values are adjusted β [95% CI] also adjusted for variables related to study design (region, recruitment wave and maternity size).

Supplementary table 3. Sensitivity analysis with multiple imputations: multivariable multinomial logistic regression analysis explaining clusters (identified by HAC) by family characteristics all together (N=8.922).

	B2'- intermediate BF, late CF, late unmixed foods, infrequent of main food groups	B3'- long BF, intermediate CF, early unmixed foods	B4'- short BF, early CF, very early sweetened beverages & fruit juices	B5'- short BF, early CF, very early cow's milk
Maternal age (years)				
< 25	0.83 [0.64 ; 1.08]	1.05 [0.82 ; 1.34]	1.56 [1.27 ; 1.92]	1.66 [1.31 ; 2.09]
25–29	0.89 [0.79 ; 1.01]	1.00 [0.88 ; 1.13]	0.94 [0.84 ; 1.05]	0.94 [0.83 ; 1.08]
30–34	1 [Ref]	1 [Ref]	1 [Ref]	1 [Ref]
≥ 35	1.34 [1.16 ; 1.54]	1.10 [0.96 ; 1.27]	0.97 [0.84 ; 1.11]	0.83 [0.7 ; 0.97]
Maternal education level				
Up to lower secondary	1.04 [0.69 ; 1.55]	0.71 [0.48 ; 1.05]	1.45 [1.01 ; 2.06]	1.22 [0.80 ; 1.85]
Upper secondary	1.19 [1.02 ; 1.38]	0.77 [0.66 ; 0.90]	1.38 [1.20 ; 1.59]	1.43 [1.22 ; 1.69]
Intermediate	1.02 [0.88 ; 1.19]	1.02 [0.88 ; 1.19]	1.15 [1.00 ; 1.33]	0.98 [0.82 ; 1.17]
3-year university degree	0.80 [0.69 ; 0.94]	1.22 [1.05 ; 1.42]	0.66 [0.57 ; 0.78]	0.72 [0.60 ; 0.87]
At least 5-year university degree	1 [Ref]	1 [Ref]	1 [Ref]	1 [Ref]
Family income (€/month/consumption unit)				
≤750	1.32 [0.94 ; 1.86]	2.05 [1.51 ; 2.77]	1.54 [1.13 ; 2.10]	1.81 [1.30 ; 2.51]
751–1111	1.03 [0.84 ; 1.27]	1.33 [1.09 ; 1.61]	1.08 [0.88 ; 1.32]	1.28 [1.03 ; 1.58]
1112–1500	1.02 [0.89 ; 1.17]	1.05 [0.92 ; 1.20]	0.99 [0.87 ; 1.13]	0.96 [0.83 ; 1.12]
1501–1944				
1945–2500	1.00 [0.86 ; 1.17]	0.75 [0.64 ; 0.88]	0.82 [0.69 ; 0.97]	0.76 [0.62 ; 0.93]
>2500	0.75 [0.62 ; 0.91]	0.59 [0.48 ; 0.71]	0.76 [0.62 ; 0.94]	0.68 [0.53 ; 0.88]
Migration status	1 [Ref]	1 [Ref]	1 [Ref]	1 [Ref]
Migrant	1.05 [0.84 ; 1.30]	1.90 [1.59 ; 2.28]	1.41 [1.16 ; 1.72]	1.04 [0.81 ; 1.34]
Descendant of migrant	1.01 [0.85 ; 1.21]	0.84 [0.71 ; 0.98]	0.95 [0.81 ; 1.13]	1.05 [0.85 ; 1.29]
Majority of population				
Single motherhood				
No	1 [Ref]	1 [Ref]	1 [Ref]	1 [Ref]
Yes	0.96 [0.74 ; 1.26]	1.15 [0.9 ; 1.46]	1.27 [1.01 ; 1.59]	1.20 [0.93 ; 1.56]
Older children in the household				
No older child	1 [Ref]	1 [Ref]	1 [Ref]	1 [Ref]
One older child	1.04 [0.95 ; 1.14]	0.92 [0.84 ; 1.01]	0.85 [0.77 ; 0.94]	0.9 [0.81 ; 1.01]
At least 2 older children	1.10 [0.96 ; 1.25]	1.33 [1.18 ; 1.51]	1.04 [0.9 ; 1.20]	1.44 [1.24 ; 1.67]
Child's sex				
Boys	1 [Ref]	1 [Ref]	1 [Ref]	1 [Ref]
Girls	1.01 [0.95 ; 1.07]	0.95 [0.89 ; 1.01]	0.91 [0.86 ; 0.97]	0.89 [0.83 ; 0.96]
Child's age at maternal return to work				
Not concerned	1.11 [0.93 ; 1.32]	1.83 [1.57 ; 2.13]	1.23 [1.05 ; 1.45]	1.36 [1.13 ; 1.63]
<10 weeks	0.83 [0.72 ; 0.95]	0.57 [0.49 ; 0.67]	1.02 [0.89 ; 1.17]	0.85 [0.72 ; 1.01]
10–13 weeks	1 [Ref]	1 [Ref]	1 [Ref]	1 [Ref]
3 to <6 months	0.97 [0.85 ; 1.1]	0.89 [0.78 ; 1.02]	0.79 [0.68 ; 0.93]	0.8 [0.67 ; 0.95]
After 6 months	1.21 [1.05 ; 1.39]	2.01 [1.76 ; 2.29]	1.08 [0.93 ; 1.26]	1.2 [1.01 ; 1.42]

Reference group: B1'- intermediate BF, intermediate CF, no sweetened beverages & fruit juices. Values are OR [95% confidence interval] adjusted on study design variables (region, recruitment wave and maternity size).

Supplementary table 4. Sensitivity analysis with weighted data: Multivariable multinomial logistic regression explaining clusters (identified by HAC) by family characteristics all together (N=7.543).

	B2'- intermediate BF, late CF, late unmixed foods, infrequent of main food groups	B3'- long BF, intermediate CF, early unmixed foods	B4'- short BF, early CF, very early sweetened beverages & fruit juices	B5'- short BF, early CF, very early cow's milk	P value
Maternal age (years)					<0.001
< 25	0.68 [0.65-0.71]	1.03 [0.98-1.07]	1.93 [1.86-2.00]	2.73 [2.62-2.84]	
25–29	0.85 [0.83-0.87]	1.14 [1.12-1.17]	1.31 [1.28-1.34]	1.32 [1.28-1.36]	
30–34	1 [ref]	1 [Ref]	1 [Ref]	1 [Ref]	
≥ 35	1.18 [1.15-1.21]	1.24 [1.21-1.27]	1.35 [1.32-1.39]	1.25 [1.21-1.29]	
Maternal education level					<0.001
Up to lower secondary	1.43 [1.35-1.52]	0.43 [0.40-0.45]	2.17 [2.05-2.30]	0.90 [0.84-0.97]	
Upper secondary	1.29 [1.25-1.33]	0.55 [0.53-0.57]	2.37 [2.30-2.45]	2.25 [2.16-2.34]	
Intermediate	0.98 [0.95-1.00]	0.69 [0.67-0.71]	1.68 [1.63-1.73]	1.28 [1.23-1.34]	
3-year university degree	0.82 [0.80-0.85]	0.85 [0.83-0.88]	0.99 [0.96-1.03]	1.05 [1.00-1.09]	
At least 5-year university degree	1 [ref]	1 [Ref]	1 [Ref]	1 [Ref]	
Family income (€/month/consumption unit)					<0.001
≤750	1.12 [1.06-1.18]	2.8 [2.67-2.94]	1.47 [1.41-1.55]	1.35 [1.28-1.43]	
751–1111	0.97 [0.94-1.01]	1.92 [1.85-1.99]	0.79 [0.77-0.82]	1.32 [1.27-1.37]	
1112–1500	0.98 [0.96-1.01]	1.31 [1.27-1.34]	0.90 [0.88-0.92]	0.97 [0.94-1.00]	
1501–1944	1 [ref]	1 [Ref]	1 [Ref]	1 [Ref]	
1945–2500	1.03 [1.00-1.06]	0.97 [0.94-1.00]	0.80 [0.78-0.83]	0.81 [0.78-0.85]	
>2500	0.72 [0.70-0.75]	0.72 [0.70-0.75]	0.80 [0.77-0.83]	0.64 [0.61-0.68]	
Migration status					<0.001
Migrant	1.16 [1.12-1.20]	2.92 [2.84-3.01]	1.52 [1.47-1.57]	1.00 [0.96-1.04]	
Descendant of migrant	1.14 [1.10-1.18]	1.50 [1.45-1.55]	1.50 [1.45-1.55]	1.25 [1.20-1.30]	
Majority of population	1 [ref]	1 [Ref]	1 [Ref]	1 [Ref]	
Single motherhood					<0.001
No	1 [ref]	1 [Ref]	1 [Ref]	1 [Ref]	
Yes	0.96 [0.90-1.03]	0.88 [0.82-0.93]	1.88 [1.78-1.98]	1.18 [1.11-1.26]	
Older children in the household					<0.001
No older child	1 [ref]	1 [Ref]	1 [Ref]	1 [Ref]	
One older child	1.20 [1.17-1.22]	1.12 [1.09-1.14]	0.70 [0.68-0.72]	1.26 [1.23-1.29]	
At least 2 older children	1.22 [1.18-1.26]	1.62 [1.57-1.67]	0.85 [0.82-0.88]	2.20 [2.12-2.28]	
Child's sex					<0.001
Boys	0.98 [0.96-0.99]	1.13 [1.10-1.15]	1.25 [1.23-1.28]	1.30 [1.27-1.32]	
Girls	1 [ref]	1 [Ref]	1 [Ref]	1 [Ref]	
Child's age at maternal return to work					<0.001
Not concerned	1.22 [1.19-1.26]	3.31 [3.21-3.42]	1.47 [1.43-1.51]	1.72 [1.66-1.78]	
<10 weeks	0.77 [0.75-0.80]	1.00 [0.96-1.04]	1.01 [0.98-1.04]	0.93 [0.90-0.97]	
10–13 weeks	1 [ref]	1 [Ref]	1 [Ref]	1 [Ref]	
3 to <6 months	0.93 [0.90-0.95]	1.65 [1.59-1.70]	0.76 [0.74-0.79]	0.79 [0.76-0.82]	
After 6 months	1.21 [1.18-1.25]	3.31 [3.21-3.42]	1.16 [1.12-1.20]	1.28 [1.24-1.33]	

Reference group: B1'- intermediate BF, intermediate CF, no sweetened beverages & fruit juices. Values are OR [95% CI] adjusted on study design variables (region, recruitment wave and maternity size).
